# Importance of dose-schedule of 5-aza-2'-deoxycytidine for epigenetic therapy of cancer

**DOI:** 10.1186/1471-2407-8-128

**Published:** 2008-05-02

**Authors:** Maryse Lemaire, Guy G Chabot, Noël JM Raynal, Louise F Momparler, Annie Hurtubise, Mark L Bernstein, Richard L Momparler

**Affiliations:** 1 Département de pharmacologie, Université de Montréal, Centre de recherche pédiatrique, Service d'Hématologie-Oncologie, Hôpital Sainte-Justine, 3175 Côte Sainte-Catherine, Montréal, Québec H3T 1C5, Canada; 2 INSERM U640-CNRS UMR8151, Laboratoire de pharmacologie chimique et génétique, Faculté de Pharmacie, Université Paris V, F75006 Paris, France; 3 Hematology-Oncology, IWK Health center, 5850/5980 University Avenue, Halifax, Nova Scotia, B3K 6R8, Canada

## Abstract

**Background:**

The inactivation of tumor suppressor genes (TSGs) by aberrant DNA methylation plays an important role in the development of malignancy. Since this epigenetic change is reversible, it is a potential target for chemotherapeutic intervention using an inhibitor of DNA methylation, such as 5-aza-2'-deoxycytidine (DAC). Although clinical studies show that DAC has activity against hematological malignancies, the optimal dose-schedule of this epigenetic agent still needs to be established.

**Methods:**

Clonogenic assays were performed on leukemic and tumor cell lines to evaluate the *in vitro *antineoplastic activity of DAC. The reactivation of TSGs and inhibition of DNA methylation by DAC were investigated by reverse transcriptase-PCR and Line-1 assays. The *in vivo *antineoplastic activity of DAC administered as an i.v. infusion was evaluated in mice with murine L1210 leukemia by measurement of survival time, and in mice bearing murine EMT6 mammary tumor by excision of tumor after chemotherapy for an *in vitro *clonogenic assay.

**Results:**

Increasing the DAC concentration and duration of exposure produced a greater loss of clonogenicity for both human leukemic and tumor cell lines. The reactivation of the TSGs (*p57KIP2 *in HL-60 leukemic cells and *p16CDKN2A *in Calu-6 lung carcinoma cells) and the inhibition of global DNA methylation in HL-60 leukemic cells increased with DAC concentration. In mice with L1210 leukemia and in mice bearing EMT6 tumors, the antineoplastic action of DAC also increased with the dose. The plasma level of DAC that produced a very potent antineoplastic effect in mice with leukemia or solid tumors was > 200 ng/ml (> 1 μM).

**Conclusion:**

We have shown that intensification of the DAC dose markedly increased its antineoplastic activity in mouse models of cancer. Our data also show that there is a good correlation between the concentrations of DAC that reduce *in vitro *clonogenicity, reactivate TSGs and inhibit DNA methylation. These results suggest that the antineoplastic action of DAC is related to its epigenetic action. Our observations provide a strong rationale to perform clinical trials using dose intensification of DAC to maximize the chemotherapeutic potential of this epigenetic agent in patients with cancer.

## Background

The inactivation of tumor suppressor genes (TSGs) by epigenetic events, such as aberrant DNA methylation, plays an important role in oncogenesis [1,2]. Since epigenetic changes are reversible, they are promising targets for chemotherapy using agents that inhibit DNA methylation [3,4]. 5-Aza-2'-deoxycytidine (decitabine, dacogen, DAC) is a potent demethylating agent [5], that has been shown to reactivate TSGs [3] silenced by promoter DNA methylation [1,2]. In preclinical studies DAC was shown to have potent antileukemic activity in the mouse model [6-8]. In addition DAC showed potent antitumor activity against a human tumor xenograft [9]. Its mechanism of action is related to the induction of terminal differentiation, senescence or apoptosis, resulting in an irreversible loss of proliferative potential [10-12].

Clinical trials in patients with hematological malignancies have shown that both low and intermediate doses of DAC can produce responses [13-18]. In patients with solid tumors, low dose DAC produced low response rates [19-21], whereas in a pilot study high doses showed promising activity [22]. Indeed, the dose-schedules of DAC used in the clinical studies were very different, and it appears that the optimal dose still needs to be refined in order to fully exploit its chemotherapeutic potential.

In order to clarify the dose-schedule of DAC as a guide to clinical trials, we have evaluated the antineoplastic activity of DAC both *in vitro *and in animal models. We have observed that DAC reduced the *in vitro *clonogenic potential in a dose-dependent manner of both human and murine leukemia and tumor cell lines. The *in vitro *antineoplastic action of DAC correlated well with its inhibition of DNA methylation and reactivation of TSG in human neoplastic cells. We also observed a correlation between *in vitro *and *in vivo *concentrations needed to produce optimal responses in mice with L1210 leukemia or with EMT6 tumor. These observations may be useful in the design of optimized dose-schedules of DAC for future clinical trials aimed at evaluating its full chemotherapeutic potential in patients with cancer.

## Methods

### Chemicals

DAC (molecular weight 228.2) was obtained from A. Piskala, Institut of Organic Chemistry and Biochemistry, Czechoslovak Academy of Science, Prague, Czeckoslovakia. DAC was dissolved in sterile phosphate buffer saline pH 6.8 (PBS) solution and stored at -70°C to prevent decomposition.

### Cell culture

The mouse lymphoid leukemia cell line L1210 was obtained from Dr. T. Khwaja (University of Southern California, Los Angeles). These cells were maintained in RPMI-1640 medium (Canadian Life Technologies, Burlington, Ontario) with 5% heat-inactivated fetal calf serum (WISENT, St-Bruno, Quebec) and with 6 μM of 2-mercaptoethanol. The mouse mammary tumor cell line EMT6 was obtained from spontaneous mammary tumor of BALB/c mouse [23]. These cells were maintained in MEM F-14 medium (Canadian Life Technologies) with 10% heat-inactivated fetal calf serum and with 25 units/ml of penicillin, 25 μg/ml of streptomycin and 0.1 mg/ml of calcium chloride. The doubling time of both murine cell lines was ~12 h. The human acute promyelocytic leukemic cells HL-60 (ATCC, USA) and the lung carcinoma cells Calu-6 were maintained in RPMI-1640 medium (Canadian Life Thecnologies) with 10% heat-inactivated foetal calf serum (WISENT). The doubling times of the HL-60 and Calu-6 cells were 16 and 30 h, respectively. All cells lines were maintained at 37°C with 5% CO_2_.

### Clonogenic assays

A 5 ml aliquot of HL-60 or L1210 (10^4 ^cells/ml) in log growth phase were placed in 25 cm^2 ^tissue-culture flasks. The indicated concentrations of DAC were added. The flasks were incubated at 37°C and at the indicated times, an aliquot was removed for counting with a Model Z Coulter Counter. An aliquot of 100 cells was placed in 2 ml of 0.3% soft agar RPMI 1640 medium containing 20% serum for HL-60 (Table 1) or 10% serum plus 6 μM 2-mercaptoethanol (Sigma) for L1210 cells (Table 3). For EMT6 tumor, 5 ml (200 cells) were placed in a Petri dish and incubated for 4 h. Different concentrations of DAC were then added for 18 h exposure (Table 3). The drug was removed and the cells were placed in drug-free medium. For Calu-6 lung carcinoma cells, 2 ml (100 cells) were plated in 6-well dishes and incubated for 24 h prior to addition of different concentrations of DAC (Table 2). The drug was removed after the different exposure times and the cells were placed in drug-free medium. The number of colonies (> 500 cells) was counted after 7 days of incubation for both L1210 and EMT6 cells (Table 3), and after 14 days of incubation for HL-60 (Table 1) and Calu-6 cells (Table 2). The cloning efficiency of all the cells was in the range of 60–75%.

**Table 1:** Effect of different concentrations and exposure times of DAC on loss of clonogenicity of human HL-60 myeloid leukemic cells.

	Loss of clonogenicity (%)			
DAC Concentration ng/ml (μM)	Exposure time			
	2 h	6 h	24 h	48 h
10 (0.044)	11.8 ± 9.8^a^	43.9 ± 10.2^a^	44.9 ± 15.9^a^	69.0 ± 19.9^a^
20 (0.088)	ND	ND	56.1 ± 19.2	ND
100 (0.44)	43.9 ± 7.8	74.0 ± 9.8	83.9 ± 4.9	99.7 ± 0.8
200 (0.88)	56.4 ± 14.8	77.2 ± 10.7	99.2 ± 2.1	100
1,000 (4.4)	61.3 ± 14.7	89.3 ± 3.8	ND	100

^a^Mean values ± S.D., n ≥ 4

**Table 2:** Effect of different concentrations and exposure times of DAC on loss of clonogenicity of human Calu-6 lung carcinoma cells.

	Loss of clonogenicity (%)			
DAC Concentration ng/ml (μM)	Exposure time			
	4 h	8 h	24 h	48 h
1 (0.0044)	ND	ND	16.4 ± 9.1^a^	10.3 ± 5.9^a^
10 (0.044)	25.5 ± 15.6^a^	20.1 ± 15.2^a^	30.7 ± 11.4	22.5 ± 19.4
100 (0.44)	59.8 ± 20.2	71.1 ± 9.3	83.9 ± 14.2	71.6 ± 11.1
1,000 (4.4)	82.4 ± 10.2	83.4 ± 1.1	98.0 ± 4.2	100 ± 0

^a^Mean values ± S.D., n ≥ 3

**Table 3:** Effect of different concentrations of DAC on loss of clonogenicity by L1210 murine lymphoblastic leukemia, or by EMT6 murine mammary tumor cells^a^

DAC Concentration ng/ml (μM)	L1210 Loss of clonogenicity (%)	EMT6 Loss of clonogenicity (%)
1 (0.0044)	25.3 ± 12.4^b^	-
10 (0,044)	41.4 ± 8.8	12.3 ± 5.5^b^
100 (0.44)	98.6 ± 1.3	69.3 ± 10.3
1,000 (4.4)	100	75.0 ± 10.8
10,000 (44)	-	99.2 ± 0.3

^a^18 h exposure to DAC.

^b^Mean values ± S.D., n ≥ 3.

### Isolation of RNA and RT-PCR

**Figure 1:**
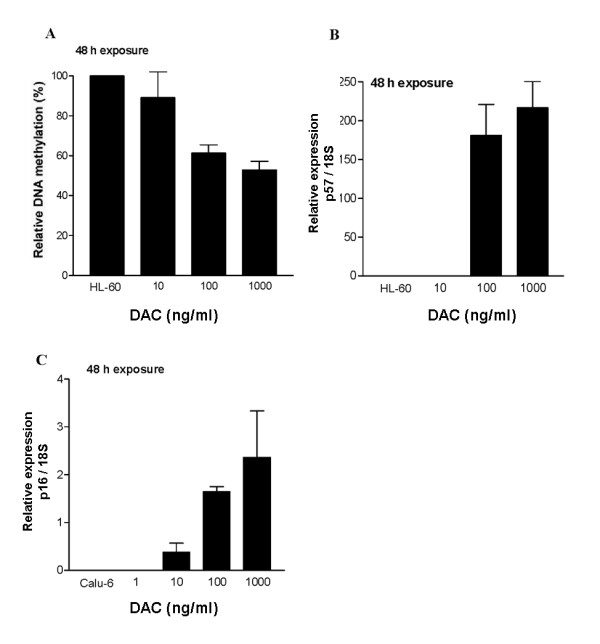
**Effect of DAC concentration on DNA methylation and reactivation of p57 and p16 tumor suppressor genes**. **A), **HL-60 cells were treated with the indicated concentrations of DAC for 48 h. Total DNA was isolated at 72 h and the LINE-1 element gene methylation status was analyzed by COBRA assay. **B), **HL-60 cells were treated with the indicated concentrations of DAC for 48 h and total RNA was isolated at 72 h. Gene expression of 18 S ribosomal RNA gene and p57 were analyzed by RT-PCR. The amount of DNA amplified during the exponential phase of PCR was analyzed by quantification of amplified DNA by an Agilent 2100 Bioanalyzer. The control cells are HL-60 with no drug treatment. Control vs DAC 100 ng/ml or 1,000 ng/ml p < 0.001. **C), **Calu-6 cells were treated with the indicated concentrations of DAC for 48 h. Total RNA was isolated at 72 h and 18 S ribosomal RNA gene and p16 gene expression analyzed by RT-PCR. The amount of DNA amplified during the exponential phase of PCR was analyzed by quantification of amplified DNA by an Agilent 2100 Bioanalyzer. For the quantitative detection of gene expression, we used 18S as the reference to normalize the data. The control cells were Calu-6 with no drug treatment. Control vs DAC 100 ng/ml p < 0.01. Data are mean values ± S.D., n ≥ 3.

In order to study the epigenetic effect of DAC, the reactivation of the tumor suppressor genes p57CDKN1C (p57) (GenBank accession number NM_000076) [8] on HL-60 cells and p16CDKN2A (p16) (GenBank accession number NM_058197) [24] on Calu-6 cells was evaluated. HL-60 leukemic cells and Calu-6 lung carcinoma cells were treated with different concentrations of DAC for 48 h (Fig. 1). Total RNA was isolated 24 h after drug removal using RNeasy Mini Kit (Qiagen, Mississauga, Ontario, CA). For cDNA synthesis, RNA was reverse-transcribed as previously described [8]. PCR amplifications were performed using HotStar Taq Polymerase (Qiagen) and specific primers spanning different exons for human 18S ribosomal RNA (18S) (Gen Bank accession number X03205) [8], p57 [8] and p16 [25]. The 18S was amplified as an internal control. Samples were amplified in a Whatman Biometra T gradient thermocycler (Göttingen, Germany) under the following conditions (previously described for 18S and p57 [8]). For p16, 15 min at 95°C to activate Taq polymerase, denaturation at 94°C for 45 s, annealing at 59°C for 30 s and extension at 72°C for 30 s, for 5 cycles. Then, the annealing temperature was lowered by 2°C for an additional 35 cycles. The length or the PCR products were 110, 167 and 117 bp for 18S, p57 and p16 respectively. For each gene, the number of cycles was determined during the exponential phase of DNA amplification. The PCR products were electrophoresed on 2% agarose gel and detected by ethidium bromide staining. For quantitative detection of gene expression, we used the 18S as the reference standard to normalize the data. The concentration of the amplified DNA was determined by Agilent 2100 Bioanalyzer (Palo Alto, CA). This instrument uses a very sensitive capillary electrophoresis and fluorescent detection to measure the concentration and size of DNA from a sample size of only 1 μl (Fig. 1B and 1C).

### Isolation of DNA and bisulfite treatment

In order to study the global methylation status of the genome of the HL-60 leukemic cells, the LINE-1 (GenBank accession number AF148856) assay was used [26]. The specific methylation status of the promoter of the p57 gene (GenBank accession number NC_000011) has also been studied in these cells. HL-60 cells were treated with different concentrations of DAC (10, 100 and 1000 ng/ml or 0.044, 0.44 and 4.4 μM) for 48 h. Genomic DNA was isolated 24 h after drug removal using DNeasy Tissue Kit (Qiagen). Genomic DNA was then treated with bisulfite [8]. This bisulfite treatment converts unmethylated cytosine residues to uracil, whereas 5-methylcytosine residues remain unchanged.

### Quantitation of DNA methylation by LINE assay

Analysis of changes in DNA methylation after DAC treatment were evaluated by the method used for the LINE-1 assay [26]. PCR was performed on bisulfite treated DNA using specific primers for either LINE-1 (sense 5'-TTA GGG AGT GTT AGA TAG TGG-3' and antisense 5'-ATA CCC TAC CCC CAA AAA TAA AAC-3' (primer sequences were designed by Allen S. Yang, personal communication). The PCR conditions were the same as RT-PCR for 18S, except the annealing temperature was 53°C and the total number of cycles was 35. The PCR products were then purified using the DNA Clean and Concentrator Kit (Zymo Research, CA, USA) and digested with *Hinf I *enzyme (New Englend BioLabs, MA, USA) according to manufacturer's protocols. *Hinf I *will only cut the repetitive LINE-1 elements that were originally methylated, leading to a 53 and a 398 bp fragmentation product that can be quantified. The samples were placed on a 2% agarose gel, detected by ethidium bromide staining and their concentrations were evaluated by the Agilent 2100 Bioanalyzer.

### Chemotherapy in mice

**Figure 2:**
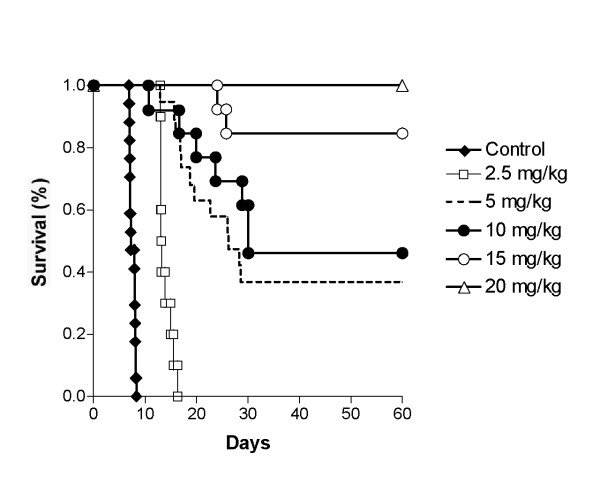
**Effect of DAC on the survival time of mice with L1210 lymphoid leukemia**. CD2F_1 _mice received 1 × 10^4 ^L1210 leukemic cells on day 1. Chemotherapy was started on day 2 with the indicated doses of DAC for 18 h i.v. infusion. The survival time of mouse was monitored. Control: n = 10 ; DAC 2.5 mg/kg : n = 10 ; DAC 5 mg/kg : n = 19 ; DAC 10 mg/kg : n = 13 ; DAC 15 mg/kg : n = 12 ; DAC 20 mg/kg : n = 3.

BALB/c × DBA/2 (hereafter called CD2F_1_) male mice 24–28 g were obtained from Taconic Biotechnology (Germantown, NY, USA). The animal committee approved the experimental protocols and the animals were handled in accordance with institutional guidelines. Transplantation of L1210 leukemic cells was performed by weekly i.p. injections of 10^4 ^cells in 0.1 ml of RPMI-1640 medium into CD2F_1 _mice. Seven days later, the ascitic fluid was obtained and a cell count of the leukemic cells was performed with a hemocytometer. For chemotherapy of leukemia, the mice were injected i.v. with 0.1 ml of L1210 (10^4 ^cells) [6]. DAC was dissolved in PBS and sterilized by 0.2 μm filtration (Fig. 2). For i.v. infusion administration, a Harvard infusion pump was used at a flow rate of 0.22 ml/h via a 25-gauge needle attached to a winged infusion set into the lateral tail vein. Mice were placed in a restrainer cage during drug treatment [8]. The survival time of each group of leukemic mice was monitored and the increase in life span (ILS) calculated (Fig. 2). Mice that survived > 60 days after drug treatment were classified as long-term survivors.

### *In vitro *clonogenic assay of tumors after *in vivo *chemotherapy [27]

For the transplantation of EMT6 mammary tumor, the tumor was removed by surgery and cut into small pieces under sterile conditions. A tumor fragment of ~1 mm diameter was injected s.c. Transplantations were performed every two weeks. CD2F_1 _mice with EMT6 tumors received DAC when the tumors reached a weight of ~200 mg (14 ± 2 days after s.c. injection of EMT6 cells). Mice were administered the i.v. infusion of DAC at a flow rate of 0.25 ml/h at 1.67 mg/kg/h for different infusion times or a single 18 h i.v. infusion at different doses. Tumors were then removed, cut and washed with medium. Cells were trypsinized and filtered (37 μm) to remove tumor fragments. The single cell suspension was centrifuged, washed with medium and counted. Cells were then plated in Petri dishes at a density of 500 and 1000 cells/dish and 14 days later, colonies were fixed, stained with Giemsa and counted. The cloning efficiency was ~10%.

### Pharmacokinetic analysis

Mice received DAC by i.v. infusion for 4 h to reach a steady state concentration at the indicated rate of infusion corresponding to different doses levels (Table 4). The mice were anesthetized with carbon dioxide. At 1–2 min post-infusion, blood samples were obtained by cardiac puncture using a syringe flushed with heparin. The blood samples were placed on ice and centrifuged at 10,000 × g for 5 min. The supernatant was obtained and used for bioassay as described previously [22]. The bioassay measures the growth inhibition of L1210 leukemic cells after 48 h incubation. Different dilutions of the plasma were added to the medium and the growth inhibition determined. The plasma concentration of DAC was estimated from the standard curve (not shown) with known concentrations of this analog. The experiment was repeated twice and performed in duplicates.

**Table 4:** Pharmacokinetics of DAC in mice.

Total dose^a ^(mg/kg)	Rate infusion (mg/kg/h)	Plasma level ng/ml (μM)
5	0.278	80 ± 0.3^b ^(0.35)
10	0.556	190 ± 0.5 (0.83)
20	1.11	610 ± 0.9 (2.68)

^a^18 h i.v. infusion of DAC. Blood sample was obtained at 4 h.

^b^Mean values ± S.D., n ≥ 3.

### Statistical considerations

For statistical analysis one-way ANOVA was performed. The p-value was evaluated accordingly to Tukey's method [28]. A value p < 0.05 was taken to indicate statistical significance. The data represented the mean values ± S.D. for n ≥ 3.

## Results

Clonogenic assays were performed on human leukemic and tumor cell lines to facilitate the translation of in vitro data to the design of a protocol for a clinical trial. Human HL-60 myeloid leukemic cells were exposed to different concentrations of DAC for different exposure times (Table 1). DAC produced a loss of clonogenicity in a time- and a dose-dependent manner. For a 24 h exposure, the concentration that produced 50% loss of clonogenicity (IC50) value was between 10 and 20 ng/ml (0.044 and 0.088 μM), whereas 200 ng/ml (0.88 μM) produced almost 100% in loss of clonogenicity.

Similarly, Calu-6 lung carcinoma cells were exposed to different concentrations of DAC for different exposure times (Table 2). DAC produced a loss of clonogenicity in a dose- and a time-dependent manner. For the 4 and 8 h exposure times, the IC50 value was in the range of 100 ng/ml (0.44 μM). Compared to HL-60 leukemic cells, higher concentrations and longer exposure times were required to achieve a loss of clonogenicity of approximately > 90%.

In order to fully understand the antineoplastic activity of DAC, we evaluated its epigenetic action at different concentrations on human leukemic and tumor cell lines. The global DNA methylation status of HL-60 leukemic cells was monitored by tracking the amount of methylated LINE-1 element [26] (Fig. 1A). After a 48 h exposure, 100 ng/ml (0.44 μM) of DAC reduced the global methylation status of HL-60 by 40% (p < 0.001, compared to untreated cells), which seems to be sufficient to inhibit almost 100% of the clonogenic potential (Table 1). Lower concentration of DAC (10 ng/ml; 0.044 μM) did not produce a significant loss in global DNA methylation (p > 0.05, compared to untreated cells) in HL-60 cells. Similar results were obtained from the examination of the tumor suppressor gene p57, reported to be methylated in HL-60 cell lines [8]. HL-60 cells exposed to 100 ng/ml (0.44 μM) for 48 h showed re-expression of p57 mRNA (Fig. 1B). These data correlate well with the concentration required to significantly decrease the promoter methylation of p57 as determined by the methylation-specific PCR assay [8] (data not shown). When HL-60 cells are exposed to lower concentrations of DAC (10 ng/ml; 0.044 μM), DNA hypomethylation was not detected, even though that concentration could inhibit approximately 70% of the clonogenicity of these cells (Table 1).

We next investigated the re-expression of p16 mRNA on Calu-6 lung carcinoma cells exposed to different concentrations of DAC (Fig. 1C), because p16 has been reported to be completely repressed by DNA methylation in these cells [24]. The observed re-expression of p16 was found to be time- and dose-dependent, with 100 ng/ml **(**0.44 μM) required to achieve a significant re-expression at 24 h (data not shown) and 48 h (Fig. 1C). These data correlated well with the efficacy in reducing the clonogenic potential of DAC, since both exposure times gave ~80% loss of clonogenicity (Table 2).

The loss of clonogenicity was determined by the reduction in colony formation after an 18 h drug exposure of the L1210 murine lymphoid leukemic cells and of the EMT6 mammary tumor cells to different concentrations of DAC (Table 3). The IC50 was ~15 ng/ml **(**0.066 μM) for L1210 and ~50 ng/ml **(**0.22 μM) for EMT6, according to calculations. In a previous study on L1210, we also observed that shorter exposure times to DAC were less effective indicating a time-dependence [6,8]. DAC produced a loss of clonogenicity in a dose-dependent manner in both cell lines, although L1210 leukemic cells are much more sensitive than the EMT6 cells. It is noteworthy that both murine and human leukemic cells were more sensitive to the antineoplastic action of 5-AZA-CdR than the solid tumor cells of either species.

CD2F_1 _mice received an i.v. injection of 10^4 ^L1210 leukemic cells on day 1 and chemotherapy was started on day 2. The short plasma half-life of DAC in mice of 20–30 min [29] supports the use of a continuous i.v. infusion for drug administration. We administered different doses of DAC ranging from 2.5 to 20 mg/kg (Fig. 2). The total dose of 2.5 mg/kg increased the survival time to 13.9 ± 1.3 days, an 85% increase in life span as compared to untreated mice which had a survival time of 7.5 ± 0.5 days. Doubling the dose to 5 mg/kg produced an increase in survival time to 26 days with 30% of the mice surviving more than 60 days, designated as long-term survivors in this model. Increasing the dose of DAC to 10 and 15 mg/kg produced a further increase in survival time and long-term survivors. At a total dose of 20 mg/kg of DAC, 100% of treated mice survived more than 60 days, indicating that they were all long-term survivors as described in this model (Fig. 2). The toxicity produced by the 20 mg/kg dose of DAC was minimal as indicated by the < 3 % loss in body weight (data not presented).

**Figure 3:**
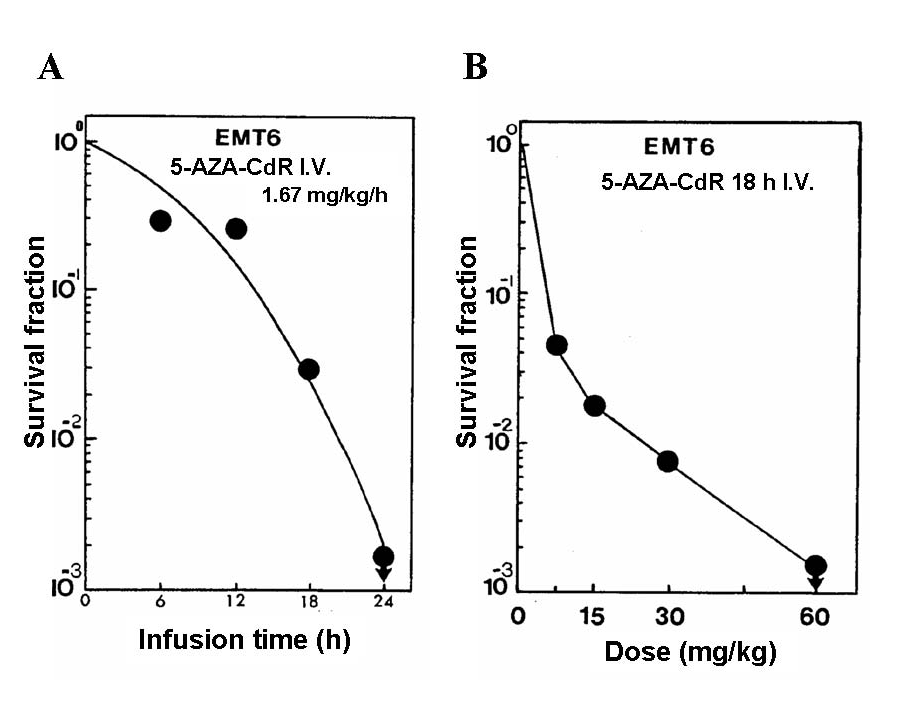
**Effect of DAC therapy on mice on the survival of EMT6 mammary tumor cells**. **A), **Mice received an i.v. infusion of DAC at 1.67 mg/kg/h for different duration (6, 12, 18 or 24 h) for total doses of 10, 20, 30 or 40 mg/kg, respectively. **B)**, Mice received an 18 h i.v. infusion at different doses of DAC (7.5, 15, 30 or 60 mg/kg). CD2F_1 _mice were injected s.c. with EMT6 tumor cells on day 1. DAC treatment as indicated was administered 14 days later. At 48 h post-treatment the tumors were removed and cells were plated in dishes for colony formation analysis. Each data point: n = 2. The cloning efficiency was about 10%.

A very sensitive method to evaluate the antitumor activity of DAC is to determine the cloning efficiency of tumor cells in vitro after an in vivo treatment. This model is more sensitive than measuring tumor volume because it is possible to detect 2 to 4 log cell kill. Mice were injected with EMT6 mammary tumor cells s.c. and the treatment with DAC was started when tumor reached a weight of approximately 200 mg. In the initial experiments, the mice received a constant rate of infusion of DAC (1.67 mg/kg/h) for different infusion times (Fig. 3A). The tumors were removed 48 h after treatment, the cells plated and colonies were counted 14 days later. The loss of clonogenicity of the tumor cells increased with the duration of the DAC infusion. A complete loss of clonogenicity was observed for the 24 h infusion at a total dose of 40 mg/kg of DAC. However, this latter dose-schedule was very toxic to the mice. In the second set of experiments, the mice received a constant 18 h infusion of DAC at different total doses of 7.5, 15, 30 and 60 mg/kg (Fig. 3B). A reduction in the survival fraction of the tumor cells was dependent on the dose of this analogue. DAC at a total dose of 30 mg/kg produced an estimated ~2 log cell kill of the tumor cells (Fig. 3B).

In a standard pharmacokinetic analysis, about 4 plasma half-lives are required for a drug to reach steady state level during a continuous i.v. infusion. The elimination half-life of DAC in plasma of mice was estimated to be 29 min [29]. For DAC it takes about 2 h to reach steady state level. In order to determine plasma level of DAC in our leukemic mouse model that produced 100% long-term survivors, we obtained blood samples after 4 h infusion time and estimated the level of this analogue using a bioassay. When DAC was infused at a rate of 1.11 mg/kg/h, which corresponds to the total dose of 20 mg/kg for 18 h infusion, its estimated plasma level was 610 ng/ml (2.68 μM) (Table 4).

## Discussion

The silencing of TSGs by aberrant DNA methylation is a frequent event in leukemogenesis and tumorigenesis. Since this epigenetic event is reversible, it provides a very interesting target for cancer therapy with DAC, a potent and specific inhibitor of DNA methylation. Recently, DAC was approved by the FDA for the treatment of the hematological malignancy, myelodysplastic syndrome [13,15,17]. However, even though some information is available concerning the dose-scheduling of DAC, the optimal dosing in humans is still not well defined. The purpose of the present preclinical study was therefore to better define the dose-schedule of DAC in order to provide data that may be useful to clinical investigators in their effort to improve the efficacy of DAC therapy in patients with cancer.

In this preclinical study we have investigated the *in vitro *and *in vivo *antineoplastic activity of DAC on leukemic and tumor cells. The objective of our study was to correlate the *in vitro *concentrations of DAC that produced a significant loss of clonogenicity with the doses that produce a potent *in vivo *antineoplastic effect in mouse cancer models. Our data clearly show that the *in vitro *loss of clonogenicity by human and murine leukemic and tumor cells increase with concentration of DAC. For HL-60 leukemic cells and Calu-6 lung carcinoma cells the loss of clonogenicity at the higher concentrations of DAC correlated with its capacity to reactivate TSGs in these cell lines. In addition, the inhibition of DNA methylation by DAC in the HL-60 leukemic cells also correlated with the loss of clonogenicity. This latter result is in accord with our previous study in mice with L1210 leukemia in which the chemotherapeutic action of DAC was correlated with its inhibition of DNA [7]. These observations further support the hypothesis that the antineoplastic action of DAC is related to its epigenetic action.

The antineoplastic action of DAC against both leukemic and tumor cells also increased with the duration of treatment. The longer exposure times allowed a greater fraction of non-S phase cells to progress into S phase, where DAC exerts its antineoplastic action [30]. Due to its S phase specificity, the duration of DAC treatment is a very important parameter that should be taken into consideration in the design of trials with this epigenetic agent in cancer patients.

In mice with L1210 leukemia, the antineoplastic activity of DAC increased markedly with the dose. The estimated steady-state plasma level of DAC was ~185 ng/ml (~0.8 μM) when administered at a total dose of 10 mg/kg as a 15-h i.v. infusion. This treatment produced ~50% long-term survivors. The *in vitro *colony assay on L1210 leukemic cells predicts a > 98% loss of clonogenicity with this concentration, indicating a good correlation between the *in vitro *and *in vivo *antileukemic action of DAC. The question arises: Is it possible to translate the observations of DAC in this mouse model of leukemia to the clinical treatment of patients with leukemia? In this regard, it is interesting to note that our data on cytosine arabinoside (cytarabine, ARA-C) in the L1210 leukemia model in mice [31] shows a good correlation with clinical studies on intensive dose ARA-C in patients with acute myeloid leukemia [32].

Although ARA-C and DAC are both deoxycytidine analogues with identical metabolism and S phase specificity, these agents have different mechanisms of action. One major concern for clinical trials using DAC dose-intensification is its hematopoietic toxicity. However, the hematopoietic toxicity seen with post-remission high dose ARA-C in leukemic patients is reversible [31]. Similarly, it should be possible to investigate more intensive dose therapy with DAC in patients with leukemia who have a good performance status, since the pharmacology would predict reversible toxicity with DAC as well [18].

The antineoplastic action of DAC against the EMT6 tumor in mice also increased markedly with dose. In this model, the tumor was excised after DAC treatment, and the cell suspension obtained for the *in vitro *clonogenic assay. We selected this model because it quantifies the survival of tumor stem cells, which should be the key target of chemotherapy [33]. DAC at total dose of 15 mg/kg produced ~2 log reduction in clonogenic survival of EMT6 tumor cells. The estimated plasma level of DAC during this infusion was 200–400 ng/ml (0.88–1.76 μM). In patients with solid tumors low dose DAC showed only minimal clinical activity [20,21]. For example, DAC (60 to 90 mg/m^2 ^total dose) administered as a continuous 72 h i.v. infusion gave steady state plasma levels in the range of 10 ng/ml (0.044 μM) [21]. In our pilot study on patients with advanced lung cancer, we used intensive dose DAC (400 to 660 mg/m^2 ^as 8 h i.v. infusion) and observed some very promising responses [22]. The mean steady-state DAC plasma level in our study was estimated to be ~650 ng/ml (2.85 μM). These preliminary clinical observations indicate that higher plasma levels of DAC are required to obtain significant antitumor activity in patients and are in accord with our preclinical data. In addition, higher plasma levels of DAC also have the potential to overcome the problem of limited penetration of the anticancer drug into the tumor as a possible cause of failure to respond to chemotherapy [34]. The antitumor activity of DAC in patients undoubtely merits further investigation in order to better refine the dose-scheduling of this epigenetic agent in patients.

## Conclusion

The *in vitro *assays indicate that the antineoplastic action of DAC on leukemic and tumor cells increase markedly with concentration. The *in vivo *antineoplastic action of DAC against murine leukemia and tumor also increase markedly with the dose. Pharmacokinetic analyses indicate that the plasma concentration of DAC that produce a potent *in vivo *antineoplastic effect is also very effective in reducing the *in vitro *clonogenicity of leukemic and tumor cells. These observations provide a strong rationale for dose intensification in future clinical studies in order to fully develop the chemotherapeutic potential of DAC in patients with cancer [35-37]. Once a very effective dose schedule for DAC is established, combination studies with other gene reactivating drugs, such as histone deacetylase inhibitors, are warranted [38-41].

## Competing interests

The authors declare that they have no competing interests.

## Authors' contributions

ML carried out the clonogenic assays, molecular studies, chemotherapy of leukemia in mice and drafted the manuscript. GGC carried chemotherapy of tumors in mice, clonogenic assays, and drafted the manuscript. NJ-MR carried out the pharmacokinetic experiments and drafted the manuscript. LFM carried out the chemotherapy of leukemia in mice. AH performed the clonogenic assays on tumors. MLB evaluated the data and drafted the manuscript. RLM conceived the study, participated in its design and coordination, and helped to draft the manuscript. All authors read and approved the final manuscript.

## Pre-publication history

The pre-publication history for this paper can be accessed here:


